# The Flexibility of Physio-Cognitive Decline Syndrome: A Longitudinal Cohort Study

**DOI:** 10.3389/fpubh.2022.820383

**Published:** 2022-06-06

**Authors:** Yi-Cheng Lin, Chih-Ping Chung, Pei-Lin Lee, Kun-Hsien Chou, Li-Hung Chang, Szu-Ying Lin, Yi-Jung Lee, Ching-Po Lin, Pei-Ning Wang

**Affiliations:** ^1^Institute of Neuroscience, National Yang-Ming Chiao-Tung University, Taipei, Taiwan; ^2^Department of Neurology, Taipei Veterans General Hospital, Taipei, Taiwan; ^3^Taipei Municipal Gan-Dau Hospital, Taipei Veterans General Hospital Branch, Taipei, Taiwan; ^4^Department of Neurology, School of Medicine, National Yang-Ming Chiao-Tung University, Taipei, Taiwan; ^5^Department of Biomedical Imaging and Radiological Sciences, National Yang-Ming Chiao-Tung University, Taipei, Taiwan; ^6^Brain Research Center, National Yang-Ming Chiao-Tung University, Taipei, Taiwan; ^7^Education Center for Humanities and Social Sciences, School of Humanities and Social Sciences, National Yang-Ming Chiao-Tung University, Taipei, Taiwan; ^8^Institute of Brain Science, National Yang-Ming Chiao-Tung University, Taipei, Taiwan; ^9^Division of Neurology, Department of Medicine, Taipei City Hospital Renai Branch, Taipei, Taiwan

**Keywords:** flexibility, reverse, cognitive decline, physical frailty, cognitive frailty

## Abstract

The mutual presence of impairments in physical and cognitive functions in older adults has been reported to predict incident disability, dementia, and mortality. The longitudinal transitions of phenotypes between these functional impairments, either individually or in combination, remain unclear. To investigate the natural course and prevalence of physical and/or cognitive impairments (CIs), we enrolled participants from a community-based population. Data were retrieved from the first (August 2011 and December 2012) and second wave (August 2013 and June 2015) of the I-Lan Longitudinal Aging Study (ILAS). All participants were classified into four groups: robust, mobility impairment (MI), CI, and physio-cognitive decline syndrome (PCDS). MI was diagnosed with weakness and/or slowness. CI was diagnosed if a subject met a cutoff below 1.5 standard deviations (SDs) of age-, sex-, and education-matched norms of any neuropsychological assessments. PCDS was combined with MI and CI. Our results showed that 38, 14, 30, and 18% of the participants were on the robust, MI, CI, and PCDS at the first wave, respectively. After 2.5 years, 17% robust, 29% MI, and 37% CI progressed to PCDS. In contrast, 33% of PCDS was reversed to non-PCDS. Predictors of conversion to PCDS included worse memory and language functions, older age, lower muscle mass, and the presence of diabetes. In PCDS, a stronger hand-grip strength, younger age, and better memory functions predicted reversion to non-PCDS status. In summary, we probed the transition of PCDS. The skeletal muscle mass/function and memory function are crucial factors associated with PCDS reversion or progression.

## Introduction

Physical frailty is a state of vulnerability characterized by reduced muscle strength, endurance, slowness, and reduced physiologic reserve (1), which affects 11–14% of people aged 65 years and older and predicts falls, disability, institutionalization, mortality (2, 3), and cognitive impairment [CI; (4–7)]. A meta-analysis study has shown that the risk for dementia was higher among those with the co-occurrence of physical frailty and CI than among those with CI alone (7).

The co-occurrence of impairments in physical and cognitive functions is clinically common, and several terms have been proposed for this specific phenotype, such as cognitive frailty and motoric cognitive risk (MCR) syndrome. The major difference between these terms is the operational definition of impairments in physical and cognitive functions. Our research group defined this unique phenotype as physio-cognitive decline syndrome (PCDS) (8).

The operational definition of PCDS was based on the findings of our previous cohort studies. We found that the mobility components of frailty (slowness and weakness cluster) were associated with poorer cognitive performance and higher mortality risk than the non-mobility components of frailty (fatigue and weight loss cluster) (9, 10). Therefore, we defined PCDS as a certain condition with slowness and/or weakness as mobility impairment (MI) as well as cognitive performance a minimum of 1.5 standard deviation (SD) below the mean for age-, sex-, and education-matched norms in any cognitive domain. We also identified the specific neuroanatomical signatures of PCDS with low skeletal muscle mass, and those with frailty had gray matter deficits in the hippocampus, cerebellum, and middle frontal gyri in the magnetic resonance imaging (MRI) study (10, 11).

Physio-cognitive decline syndrome affects 10–15% of community-dwelling older adults and deserves further research (10). The pathophysiology between CIs only and the concomitant presence of impairments in physical and cognitive functions may be different, which is still unclear. A recent post-mortem pathological study further demonstrated that the neuropathologic burden was related to frailty and mild CI, or dementia. This study showed that neuropathologic features, including β-amyloid deposition, hippocampal sclerosis, Lewy bodies, tangle density, TDP-43, cerebral amyloid angiopathy, arteriolosclerosis, atherosclerosis, and gross and chronic cerebral infarcts (12), are different from β-amyloid and tangle density in Alzheimer's dementia (13). These findings suggest that there is an extraordinary pathophysiological relationship between physical frailty and related cognitive decline, which may differ from the well-recognized neurodegenerative Alzheimer's disease (AD). The clinical outcomes of PCDS may also differ from those of mild CI or prodromal AD without physical frailty.

Hence, this study aimed to evaluate longitudinal transitions in the phenotypes of older adults with MI, CI, and PCDS to explore the potential reversibility of PCDS (1, 14–16), and to identify factors associated with phenotypic transitions using the data from the I-Lan Longitudinal Aging Study (ILAS).

## Materials and Methods

### Study Design and Participants

The ILAS was a community-based aging cohort study in I-Lan County, Taiwan, which was designed to evaluate the complex interrelationship between aging, frailty, and cognitive function (9). ILAS enrolled community-dwelling adults aged 50 years and above from I-Lan County with the following inclusion criteria: (1) inhabitants of I-Lan County, (2) aged 50 years or above, and (3) no recent plans to move to other counties. ILAS excluded people with the following conditions for participation: (1) inability to communicate and complete an interview, (2) unable to complete assessments due to poor functional status, (3) having a life expectancy <6 months due to a major illness, and (4) being institutionalized. Data retrieved for this study further excluded participants with major neuropsychiatric diseases such as dementia, stroke, brain tumor, or major depression based on self-report or assessment results. Data of the first (baseline) and second wave (follow-up) were included for analysis in the present study. All participants provided written informed consent. This study was approved by the institutional review board of the National Yang-Ming Chiao-Tung University.

### Demographic Data and Functional Assessments

Demographic information, including age, sex, years of education, body weight, and height, were collected in both first and second waves of evaluation. The medical history of each participant was assessed by trained research nurses, including diabetes mellitus (DM), hypertension, hyperlipidemia, and cardiovascular disease. According to Fried's criteria, physical frailty is defined by five components: weight loss, exhaustion, low physical activity, weakness, and slowness (17). In this study, weight loss was identified as an unintentional weight loss >5% in the past year or >3 kg in the last 3 months, and exhaustion was defined using the Center for Epidemiologic Studies Depression Scale (18). Physical activity was assessed using the International Physical Activity Questionnaire-Taiwan edition (19), and low physical activity was defined as the lowest quintile within sex. Handgrip strength was measured using a digital dynamometer (Smedlay's Dynamo Meter; TTM, Tokyo, Japan) of the dominant hand, and the best result of the three trials was recorded as the muscle strength. The 6-m usual walking speed with static start and without deceleration was used to define slowness. The lowest quintile of walking speed was defined as the cutoff for slowness, and the sex-specific lowest quintile of handgrip strength was defined as weakness. We used the appendicular skeletal muscle mass (ASM) index to represent the amount of muscles in individuals (20).

### Cognitive Function Assessment

In addition to the Chinese version of the Mini-Mental Status Examination (MMSE), all participants underwent comprehensive neuropsychological assessments across multiple cognitive domains in both first and second waves of evaluation, which included (1) verbal memory: a delayed recall in the Chinese Version Verbal Learning Test (CVVLT) (21), (2) language: Boston Naming Test (BNT) (22), and category (animal) Verbal Fluency Test (VFT) (3, 23) Visuospatial function: Taylor Complex Figure Test (CFT) (24); and (4) executive function: Clock Drawing Test (CDT) (25). All these neuropsychological assessments were culturally adapted and validated (21, 26–29).

### Definition of MI, CI, and PCDS

In this study, MI was defined as the presence of weakness and/or slowness of participants, where the cutoffs recommended by the Asian Working Group for Sarcopenia were used (30). CI was defined as 1.5 SD below the mean for age-, sex-, and education-matched norms in any cognitive domain; however, without global CI. PCDS was defined as the concomitant presence of MI and CI (10). According to the epidemiological studies on the Taiwanese population, global CI was indicated as MMSE < 24 in the well-educated participants (education years ≥6) or <14 in less-educated participants (education years <6) (31). In this case, we excluded participants who had the above conditions for possible global CI or dementia.

### Statistical Analysis

All participants in the first and second wave were classified into four clinical phenotype categories: robust, MI only, CI only, and PCDS. Continuous variables are expressed as mean ± SD and categorical variables as numbers (proportions). To compare the characteristics of study participants across the different groups (robust, MI, CI, and PCDS groups), we used the chi-squared test for dichotomous variables and one-way analysis of variance (ANOVA) for continuous variables. To explore the possible predictors for the transition of phenotypes, Tukey' test was used for *post hoc* analysis due to its sensitivity for multiple comparisons. The cumulative probability (95% confidence interval) of transitions among the four groups in a 2.5 year follow-up was calculated using the cumulative distribution function of the standard normal distribution.

To investigate the factors that influence the categorical transition of four different groups, we first used the chi-squared test for dichotomous variables and one-way ANOVA for comparisons of continuous variables of four transitioned groups in a 2.5-year follow-up in each category classified at baseline (the first wave of assessment). Variables showing statistical significance after *post hoc* analyses between those who remained in the same group and who progressed to a more severe group or reversed to a milder group were included in the following multivariate binomial logistic regression models. For example, in the robust group classified at baseline, the variables that showed statistically significant differences between those who remained in the robust group and those who progressed to the PCDS group in a 2.5 year follow-up in *post hoc* analyses were put into the multivariate binomial logistic regression model (transition vs. maintenance) to determine whether the factors were the significant predictors of PCDS transition in the robust subjects. SPSS software (version 15.0; SPSS, Inc., Chicago, IL, USA) was used for statistical analysis. All tests were two-sided, and the value of *p* < 0.05, was considered significant.

## Results

### Demographic Characteristics

In the first wave of the ILAS, data from 1,223 participants were eligible for analysis. Due to funding and administrative limitations, the second wave of the ILAS was applied to a smaller random sample using the simple random sampling method. Overall, 531 participants, aged 51–87 years, completed both the first and second wave assessments with a mean follow-up of 2.5 years. Comparisons between participants from both the waves (*n* = 531) and wave 1 only (*n* = 692) are shown in Supplementary Table 1. Participants who received assessments in both the waves were older (64.46 ± 8.58, vs. 61.61 ± 8.82, *p* < 0.001), had fewer educational years (5.88 ± 4.72 vs. 7.67 ± 5.08, *p* < 0.001), slower walking speeds (1.49 ± 0.44 vs. 1.68 ± 0.46, *p* < 0.001), a lower BNT score in language function (9.51 ± 2.93, vs. 14.69 ± 2.79, *p* < 0.001), and a lower CDT score in executive function (7.28 ± 2.59, 8.08 ± 2.53, *p* < 0.001) than those who were not selected in the second wave study.

In this study, we only included data from participants who attended both wave assessments for further analyses. Table 1 summarizes the baseline characteristics and comparisons between the groups. There are no significant differences in gender and past medical history. There are significant changes in age, education, weight, height, physical functions (walking speed and grip-strength), cognitive tests (CVVLT, BNT, VFT, CFT, and CDT), MMSE, and muscle mass index (ASM). *Post hoc* analysis showed that the MI group had significantly lower height and lower ASM compared with the robust group. In contrast, the CI group had significantly older age and weaker handgrip strength than the robust group. Notably, the PCDS group was older than the MI group and had less education and poorer performance in several cognitive domains and MMSE than the CI group.

**Table 1:** Demographic characteristics of four categories at baseline.

	**Total**	**Robust**	**MI**	**CI**	**PCDS**	***p*-Value**
*n*	531	206	75	154	96	
Sex, Female, *n* (%)	246 (46%)	91 (44%)	35 (47%)	68 (44%)	52 (54%)	0.385
Age, year	64.38 ± 8.57	62.51 ± 8.06	63.18 ± 8.52	65.94 ± 8.55^b^	66.81 ± 8.78^ce^	0.001
Education, year	5.88 ± 4.72	6.84 ± 4.88	5.89 ± 4.82	5.69 ± 4.40	4.09 ± 4.29^cf^	0.001
Weight, kg	62.79 ± 11.03	63.88 ± 11.31	61.56 ± 10.39	63.46 ± 10.02	60.36 ± 12.08^c^	0.042
Height, cm	159.03 ± 8.00	160.50 ± 8.29	157.56 ± 7.72^a^	159.72 ± 7.10	155.91 ± 7.97^cf^	0.001
**Physical function assessments**						
Walking- speed, m/s	1.49 ± 0.44	1.65 ± 0.41	1.25 ± 0.39^a^	1.56 ± 0.43^d^	1.21 ± 0.35^cf^	0.001
Grip- strength, KGs	29.44 ± 9.41	32.90 ± 8.96	26.07 ± 9.38^a^	30.31 ± 7.84^bd^	23.25 ± 8.88^cf^	0.001
**Cognitive function assessments**						
**CVVLT**						
Score	6.79 ± 2.14	7.70 ± 1.18	7.64 ± 1.19	6.04 ± 2.39^bd^	5.38 ± 2.64^cef^	0.001
Impairment, *n* (%)	91 (17.1%)	0 (0%)	0 (0%)	54 (35.1%)	37 (38.5%)	0.001
**BNT**						
Score	9.51 ± 2.93	10.92 ± 2.38	10.21 ± 2.05	8.50 ± 2.95^bd^	7.56 ± 2.88^cef^	0.001
Impairment, *n* (%)	93 (17.5%)	0 (0%)	0 (0%)	53 (34.4%)	40 (41.7%)	
**VFT**						
Score	14.81 ± 4.66	16.65 ± 4.50	15.68 ± 4.08	13.24 ± 4.39^bd^	12.70 ± 4.16^ce^	0.001
Impairment, *n* (%)	54 (10.2%)	0 (0%)	0 (0%)	38 (24.7%)	16 (16.7%)	0.001
**CFT**						
Score	30.86 ± 5.95	33.01 ± 3.43	31.88 ± 4.41	29.69 ± 6.25^bd^	27.33 ± 8.28^cef^	0.001
Impairment, *n* (%)	43 (8.1%)	0 (0%)	0 (0%)	28 (18.2%)	15 (15.6%)	0.001
**CDT**						
Score	7.28 ± 2.59	8.54 ± 1.58	8.07 ± 1.76	6.49 ± 2.72^bd^	5.25 ± 2.96^cef^	0.001
Impairment, *n* (%)	112 (21.1%)	0 (0%)	0 (0%)	63 (40.9%)	49 (51.0%)	0.001
MMSE Score	26.15 ± 3.44	27.29 ± 2.65	27.12 ± 2.40	25.71 ± 3.42^bd^	23.63 ± 4.16^cef^	0.001
**Muscle mass index**						
ASM	18.22 ± 4.07	18.84 ± 4.32	17.40 ± 3.96^a^	18.47 ± 3.76	17.14 ± 3.83^c^	0.002
**Medical history**						
HTN	41%	39%	44%	40%	45%	0.732
DM	17%	18%	21%	16%	16%	0.710
HLD	11%	12%	12%	9%	14%	0.131
CAD	4%	2%	1%	7%	3%	0.133

*ASM, appendicular skeletal muscle mass index; BNT, Boston Naming Test; CAD, cardiovascular disease; CDT, Clock Drawing Test; CFT, Taylor Complex Figure Test; CI, cognitive impairment; CVVLT, Chinese Version Verbal Learning Test; DM, diabetes mellitus; HLD, hyperlipidemia; HTN, hypertension; MI, mobility impairment; MMSE, Mini-Mental State Examination; PCDS, physio-cognitive decline syndrome; VFT, Verbal Fluency Test*.

*Data showed mean ± standard deviation (SD)*.

*Significant after post hoc analyses between groups. a: significance between MI and robust group; b: significance between CI and robust group; c: significance between PCDS and robust group; d: significance between MI and CI; e: significance between MI and PCDS; f: significance between CI and PCDS*.

*Chi-squared test for dichotomous variables and ANOVA for continuous variables, significantly (p < 0.05)*.

### The Phenotypic Transition During Follow-Up

**Figure 1:**
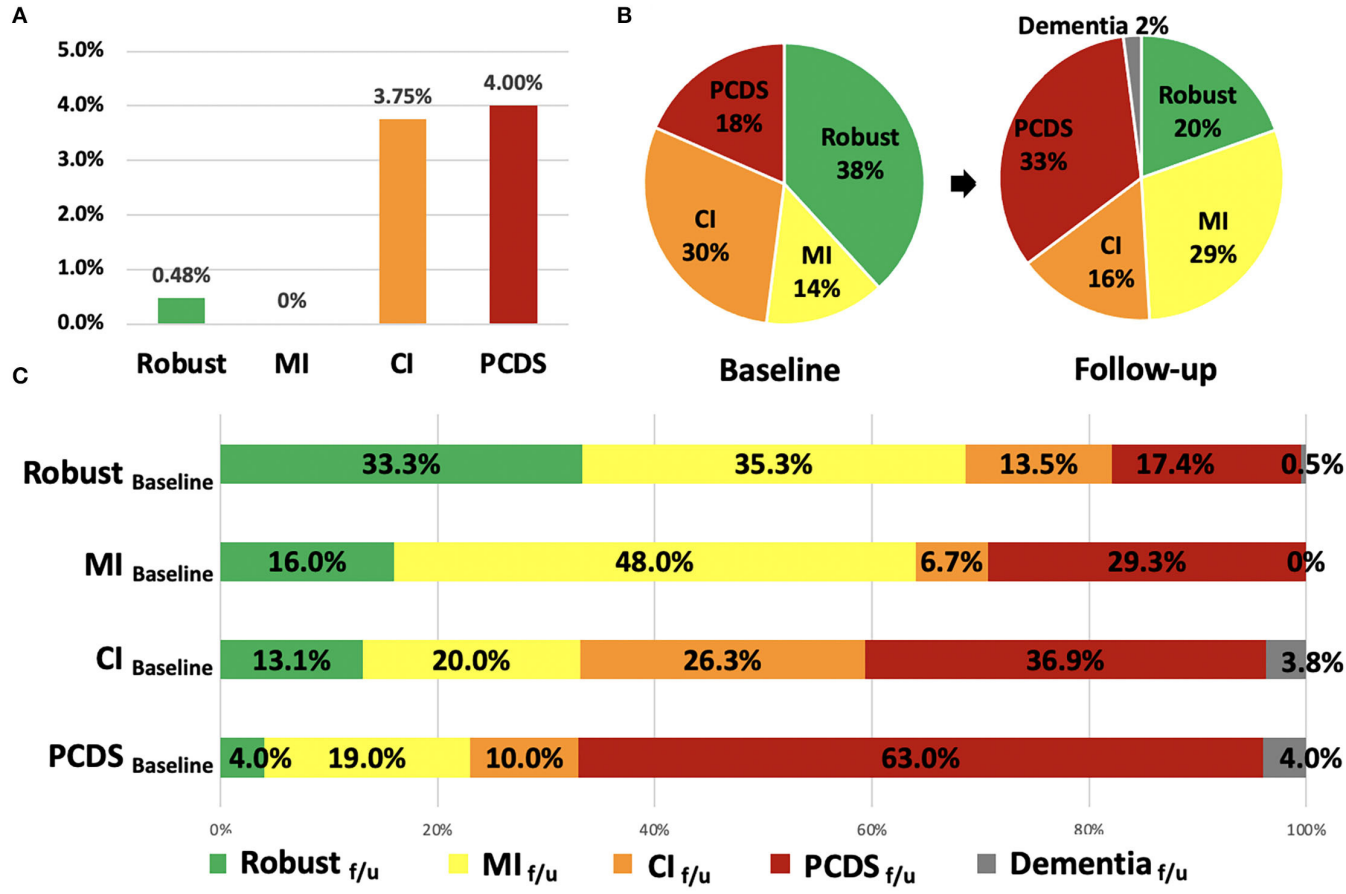
The incidence of dementia, the distribution and transitions of robust, physical frailty, CI, and PCDS groups at the first and follow-up visit. **(A)** The incidence of dementia in the four groups after 2.5 years. **(B)** The distribution of robust, physical frailty, CI, and PCDS groups at the first and follow-up visits. **(C)** The categorical transitions at the follow-up visit in four categories. CI, cognitive impairment; MI, mobility impairment; PCDS, physio-cognitive decline syndrome.

At baseline, the prevalence of robust, MI, CI, and PCDS groups was 38, 14, 30, and 18%, respectively (Figure 1B). After 2.5 years, the PCDS group had a higher risk of developing dementia (4.0%), which was similar to the CI group (3.75%, *p* = 0.889); however, it was higher than the robust group (0.48%, *p* = 0.04). However, no participant in the MI group had a high risk of dementia during follow-up (Figure 1A). As dementia is an irreversible state, this study focused on the flexibility of PCDS. Next, we examined the transition among these four groups after excluding participants who were already demented at the second wave.

In the second wave, the distribution of the four groups was 20, 29, 16, and 33%, respectively (Figure 1B). About 2% of the participants progressed to dementia after 2.5 years. More than half (63%) of the PCDS participants remained in PCDS at a follow-up. Moreover, 4.0% of PCDS participants returned to be robust, 19.0% became MI only, and 10.0% became CI only. In contrast, 17.4% of robust, 29.3% of MI, and 36.9% of CI participants progressed to PCDS in a follow-up (Figure 1C).

### Factors Associated With Phenotypic Transitions

One-way ANOVA with *post hoc* Tukey' test was used to identify variables with significant differences at baseline between each group of phenotypic transitions (Table 2, Supplementary Tables 2–4), and those variables that reached statistical significance were then entered into the multivariate binomial logistic regression model as independently associated factors (Table 3).

**Table 2:** Comparisons of the baseline physical and cognitive performance of four groups transited from participants with PCDS (*n* = 96).

	**Baseline: PCDS**				***p*-value**
2.5 years of follow-up	**Robust**	**MI**	**CI**	**PCDS**	
n	4	19	10	63	
Sex (F)	50%	58%	50%	54%	0.981
Age	60.13 ± 5.05	62.27 ± 5.76^e^	66.97 ± 6.25	68.58 ± 9.44	0.022
Education	6.75 ± 1.50	5.68 ± 4.85^e^	3.90 ± 2.85	3.48 ± 4.30	0.143
Weight	57.23 ± 9.84	60.1 ± 9.80	67.52 ± 18.89	59.50 ± 11.38	0.248
Height	157.60 ± 8.18	157.22 ± 8.10	154.48 ± 8.29	155.63 ± 7.99	0.789
Walking speed	1.02 ± 0.13	1.30 ± 0.42	1.33 ± 0.38	1.18 ± 0.32	0.251
Hang grip strength	35.75 ± 11.53^c^	24.90 ± 10.80^e^	23.60 ± 7.49	21.91 ± 7.72	0.024
CVVLT	5.00 ± 3.56	6.53 ± 1.87^e^	7.00 ± 1.49^f^	4.79 ± 2.75	0.010
BNT	10.25 ± 1.71^c^	8.79 ± 2.32^e^	6.80 ± 2.74	7.14 ± 2.95	0.031
VFT	11.50 ± 4.80	14.21 ± 3.61	12.50 ± 4.50	12.35 ± 4.21	0.347
CFT	34.75 ± 1.50^c^	29.97 ± 5.21	28.35 ± 8.85	25.91 ± 8.82	0.056
CDT	7.25 ± 1.50	6.21 ± 2.74	5.90 ± 2.77	4.73 ± 3.02	0.101
MMSE	27.00 ± 2.45^c^	25.32 ± 3.46^e^	24.00 ± 3.74	22.84 ± 4.30	0.043
ASM	17.76 ± 3.91	17.76 ± 4.31	17.52 ± 4.39	16.85 ± 3.65	0.800
HTN	0%	26%	60%	51%	0.051
DM	0%	0%	20%	21%^e^	0.143
HLD	0%	5%	10%	18%	0.458
CAD	0%	0%	0%	5%	0.666

*ASM, appendicular skeletal muscle mass index; BNT, Boston Naming Test; CAD, cardiovascular disease; CDT, Clock Drawing Test; CFT, Taylor Complex Figure Test; CI, cognitive impairment; CVVLT, Chinese Version Verbal Learning Test; DM, diabetes mellitus; HLD, hyperlipidemia; HTN, hypertension; MI, mobility impairment; MMSE, Mini-Mental State Examination; PCDS, physio-cognitive decline syndrome; VFT, Verbal Fluency Test*.

^*^Significant after post hoc analyses between groups. a: significance between the MI and robust group; b: significance between the CI and robust group; c: significance between the PCDS and robust group; d: significance between MI and CI; e: significance between MI and PCDS; f: significance between CI and PCDS.

^**^Chi-squared analysis using Tukey' test with corrected p-value, significantly (p < 0.05).

**Table 3:** Statistically significant factors affecting categorical transitions in a 2.5-year follow-up revealed by multivariate binomial logistic regression.

	**Factors**	**OR**	**95% CI**	***p*-value**
**Baseline Robust**				
Transition to:				
MI	None detectable
CI	CVVLT	0.55	0.37–0.82	0.004
PCDS	CVVLT	0.63	0.41–0.96	0.030
	VFT	0.83	0.73–0.95	0.011
**Baseline MI**				
Transition to:				
Robust	None detectable
CI	None detectable
PCDS	Age	1.13	1.04–1.22	0.004
	ASM	0.76	0.61–0.94	0.012
**Baseline CI**				
Transition to:				
Robust	Age	0.83	0.74–0.94	0.002
	BNT	1.47	1.11–1.93	0.014
MI	Age	1.25	1.12–1.38	0.0001
PCDS	DM	6.82	1.47–31.69	0.012
**Baseline PCDS**				
Transition to:				
Robust	Hand-grip strength	1.36	1.07–1.73	0.011
MI	Age	0.92	0.86–0.98	0.013
CI	CVVLT	1.57	1.05–2.35	0.032

*ASM, appendicular skeletal muscle mass index; BNT, Boston Naming Test; CI, cognitive impairment; CVVLT, Chinese Version Verbal Learning Test; DM, diabetes mellitus; MI, mobility impairment; PCDS, physio-cognitive decline syndrome; VFT, Verbal Fluency Test*.

#### Transition of Baseline Robust Group and the Predictive Factors for Transition

Compared to participants who remained robust, participants progressed to CI were older and had a lower CVVLT score in memory function at baseline, and those progressed to PCDS group had lower education years, a lower CVVLT score in memory function, and a lower VFT score in language function (Supplementary Table 2). Multivariate binomial logistic regression showed that a lower CVVLT score in memory function was a significant predictor of CI conversion (odds ratio [OR] = 0.55, *p* = 0.004), and a lower CVVLT score in memory function (OR = 0.63, *p* = 0.03) or a VFT score in language function (OR = 0.83, *p* = 0.01) were significant factors for PCDS conversion in the robust group (Table 3).

#### Transition of Baseline MI Group and the Predictive Factors for Transition

Compared to participants who remained in MI, those who progressed to PCDS were older, had fewer years of education, slower walking speed, weaker hand-grip strength, lower ASM, lower BNT and VFT scores in language function, the CDT score in executive function, and the MMSE score compared to those in the MI group (Supplementary Table 3). Only older age and lower ASM were significantly associated with PCDS conversion in MI participants (age: OR = 1.13, *p* = 0.004; ASM: OR = 0.76, *p* = 0.01) (Table 3). In the MI group, no associated factors were identified among MI to become CI.

#### Transition of Baseline CI Group and the Predictive Factors for Transition

Compared to participants who remained in CI, those who reversed CI to robust were younger and had a higher BNT score in language function (Supplementary Table 4), which remained statistically significant in the binomial regression model (age: OR = 0.83, *p* = 0.002; BNT: OR = 1.47, *p* = 0.01) (Table 3). And those who converted to MI were younger and had a higher CVVLT score in memory function at baseline than those who remained in CI (Supplementary Table 4). Further logistic regression showed that only older age was an independent factor associated with CI to MI conversion (OR = 1.25, *p* = 0.0001) (Table 3). In CI participants, those who progressed to PCDS had DM (Supplementary Table 4). Moreover, DM was an independent factor associated with progression to PCDS (OR = 6.82, *p* = 0.01) (Table 3).

#### Transition of Baseline PCDS Group and the Predictive Factors for Transition

Compared to participants who remained in PCDS, those who reversed to robust had stronger hand-grip strength (Table 2), and better hand-grip strength remained to be an independent associated factor in the binomial regression model (OR = 1.36, *p* = 0.01) (Table 3). And those who reversed PCDS to the CI group had a higher CVVLT score in memory function, and those who reversed to MI had a younger age (Table 2). Younger age was an independent associated factor for PCDS to MI, a higher CVVLT score in memory function was an independent associated factor for PCDS to CI (age: OR = 0.92, *p* = 0.01; CVVLT: OR = 1.57, *p* = 0.03) (Table 3).

## Discussion

Overall, our study demonstrated that the phenotypes of physio-cognitive decline are potentially reversible. It included 18% robust, 29% MI, and 39% CI groups that progressed to PCDS. Notably, 34.4% of the PCDS participants reversed their phenotypes into robust (4.2%), MI (19.8%), and CI (10.4%). In our studies, we probe the trajectories of PCDS. Skeletal muscle mass and mobility function are the most important factors for the phenotypic transitions of physical and CI. A lower appendicular skeletal muscle mass index (ASM) in MI participants was more likely to progress to PCDS, and PCDS participants with stronger handgrip strength, younger age, and better verbal fluency were more likely to revert to the non-PCDS status.

Mobility impairment and skeletal muscle mass loss are important in PCDS progression. These findings persisted not only in our epidemiological study but also in our basic research results. In our aging cell and animal model study, exosomal miR-29b-3p secreted by atrophic skeletal muscle impairs the development of neurons and induces neuronal senescence (32). Our previous neuroimaging study also demonstrated that individuals with physical prefrailty or frailty were present with gray matter deficits in the hippocampus, cerebellum, and middle frontal gyri (8, 11, 33). All of these evidences reinforce that phenotypic transitions in the physio-cognitive decline phenomenon are associated with a skeletal muscle–brain crosstalk.

Studies have reported that people with cognitive frailty have a poor cognitive function in some specific cognitive domains, such as working memory, verbal fluency, and processing speed (34, 35). Few studies have reported sequential changes in the specific cognitive function of PCDS (or cognitive frailty) (36, 37). Our previous study revealed that both non-memory and memory domains are associated with physical frailty (9). In this study, we also found that both memory and non-memory cognitive functions (CVVLT and VFT scores) could predict the progression from robustness to PCDS. Non-memory cognitive functions are involved earlier in physical frailty-related CI in our previous study (9). People with physical frailty tend to progress and develop PCDS during the decline of the memory function. The memory function (CVVLT score) appears to be a significant predictor of PCDS conversion in our study. In contrast, patients with PCDS with a good memory function are more likely to revert.

In our study, the PCDS group had a higher incidence of dementia (4.0%) than the robust group (0.48%). Based on our study, PCDS may be one of the main contributors to frailty-related incidental dementia instead of physical frailty alone. Our findings are in line with previous reports that CI and frailty were found to be significant risk factors for dementia instead of physical frailty (38). Alternatively, those with a combination of physical frailty and CI had a higher risk of dementia than those with physical frailty or CI alone. Therefore, CI in the MI group should be assessed for detecting PCDS. These findings also support the hypothesis that PCDS may differ from dementia in its patho-etiology (39, 40).

The criteria of original cognitive frailty are defined as physical frailty and the Clinical Dementia Rating scale 0.5 scores (41). Moreover, the following criteria further define “potentially reversible cognitive frailty” and “reversible cognitive frailty” based on the presence of objective or only subjective cognitive decline (42). Additionally, MCR was recognized as a state of concurrent physical frailty and CI. It is defined as a predementia syndrome characterized by slow gait and cognitive complaints (43, 44). Both cognitive frailty and MCR have been associated with a higher risk of incident dementia and all-cause mortality.

Compared to cognitive frailty and MCR, PCDS defines physical decline as weakness and/or slowness, but not the other components of physical frailty, and CI as objective CI in any domain. This definition is based on our previous findings that CI is more likely to be associated with MI (weakness and slowness) (9–11). MI was a good predictor of low survival rate (hazard ratio: 6.82) and poorer overall health outcomes (hazard ratio: 1.67) in our previous study (45). A recent longitudinal cohort study showed that MI was associated with a functional decline and the progression of multimorbidity, compared with the subtypes of no mobility and low physical activity (46). MI was also associated with a fast clinical decline using a data-driven approach (47). MI is an important predictor of cognitive frailty. Additionally, only subjective cognitive decline in the MCR criteria is not sufficient for CI (8). Therefore, the criteria of PCDS, including MI (weakness and/or slowness) and CI, are suitable for further studies.

Regarding comorbidities, we found that DM (48) was an independent factor for CI participants to progress to PCDS. DM has been reported to be associated with the development of frailty and dementia (49, 50). DM was at a greater risk of developing cognition impairment (49, 51); therefore, older patients with diabetes may experience CI earlier than PCDS. However, more studies are needed to confirm the pathophysiological roles of DM in the development of PCDS. Additionally, other comorbidities, including hypertension, hyperlipidemia, and cardiovascular disease, were not significantly associated with PCDS progression in our study. This might be related to the fact that most of our participants were on medications for these comorbidities. In such cases, the risk of PCDS may be low.

We have demonstrated that PCDS is a variable status with flexibility. In addition to its ability to predict poor physical and cognitive functions after 2.5 years, its flexibility was also shown in the present study. Recently, people with cognitive frailty underwent multidomain interventions, including physical, nutritional, cognitive, and psychosocial aspects, showed improvements in physical and psychosocial functions, which indicated the flexibility of cognitive frailty (52, 53). Several studies have showed factors that are associated with the reversal of frailty progression, including exercise (muscle strength training), protein supplementation, and high self-rated health (54–57). Accumulating evidences also showed the reversion from mild CI to normal cognition (58–60). Although the relationship between frailty and cognitive function impairment and how they interacted with each other are still lacking, the present study showing the factors associated with the reversion of PCDS provided clues to understand the pathophysiology of physical frailty-related CI. In addition, this study provided the nature of the flexibility of PCDS, which highlighted that early intervention is important to prevent falls, disability, institutionalization, and mortality (2, 3).

Our result suggests that, although PCDS is supposed to be a prodromal accelerated aging phenotype, it is also an important potential intervention target to prevent poor prognosis in older adults. Although in our study, there was no further intervention such as care program, policy implementations, or treatment process. Only those with chronic diseases such as hypertension, DM, or hyperlipidemia used medication. We showed that factors associated with the reversion and progression of PCDS also provided directions for interventions. We anticipate our criteria to define a high-risk PCDS group, which has the possibility of reversion for further intervention studies. Our PCDS definition supports the efficacy of a multidomain intervention to improve the function of individuals with PCDS, who are vulnerable but reversible and flexible (61–63). In addition, our study demonstrated that skeletal muscle mass and function are key factors associated with the reversion or progression of PCDS, which indicated that exercise training may be a good intervention to prevent physical and CIs (15, 52, 53, 64).

This study has some limitations. First, the participants in this study were living in rural communities and had lower educational status and good physical function, which may have overestimated the extent of CI and underestimated the extent of MI in our study population. We would need another cohort of different backgrounds to validate the present results. Second, the present longitudinal study only analyzed the follow-up data at 2.5 years. Because age-related physical or/and cognitive decline is a long-term process, we would need a longer follow-up duration to elucidate the entire disease course of PCDS. Third, this study did not record the medication used in our participants. We did not know the effect of medication on PCDS. Fourth, we did not have the data of intermediate status changes, which are important for a convertible or reversible process. Finally, we did not check for biomarkers related to degenerative dementia. According to a recent study, the potential for reversibility of cognitive frailty should be supported by the evidence of biomarkers of amyloid, tau, and neuronal damage (65). Further studies involving these biomarkers and a revised definition of cognitive frailty according to multidimensional subtyping may be needed.

In conclusion, our study showed that the phenotypes of physio-cognitive decline are potentially convertible and reversible. In the first wave, 38, 14, 30, and 18% of the participants were in the robust, MI, CI, and PCDS, respectively. After 2.5 years, 17% robust, 29% MI, and 37% CI progressed to PCDS. Skeletal muscle mass and mobility function are the most important factors for the phenotypic transitions of physical and CI. Lower ASM in MI participants was more likely to progress to PCDS, and PCDS participants with stronger handgrip strength, younger age, and better verbal fluency were more likely to revert to the non-PCDS status. We probed the transition of PCDS. Skeletal muscle mass/function and memory function are crucial factors associated with the reversion or progression of PCDS.

## Data Availability Statement

The raw data supporting the conclusions of this article will be made available by the authors, without undue reservation.

## Ethics Statement

The studies involving human participants were reviewed and approved by the Institutional Review Board of the National Yang-Ming Chiao-Tung University. The patients/participants provided their written informed consent to participate in this study.

## Author Contributions

P-NW designed the experiments. P-NW, K-HC, L-HC, and Y-JL enrolled the participants. P-LL, Y-CL, S-YL, and C-PL analyzed the data. Y-CL, C-PC, and P-NW wrote this manuscript. All authors contributed to the article and approved the submitted version.

## Funding

This work was supported by grants from the Ministry of Science and Technology, Taiwan MOST 108-2321-B-010-013-MY2 and MOST 110-2321-B-010-007, and Taipei Veterans General Hospital V108C-060.

## Conflict of Interest

The authors declare that the research was conducted in the absence of any commercial or financial relationships that could be construed as a potential conflict of interest.

## Publisher's Note

All claims expressed in this article are solely those of the authors and do not necessarily represent those of their affiliated organizations, or those of the publisher, the editors and the reviewers. Any product that may be evaluated in this article, or claim that may be made by its manufacturer, is not guaranteed or endorsed by the publisher.

## References

[1] Morley JE Vellas B van Kan GA Anker SD Bauer JM Bernabei R. Frailty consensus: a call to action. J Am Med Dir Assoc. (2013) 14:392–7. 10.1016/j.jamda.2013.03.022 23764209 PMC4084863

[2] Collard RM Boter H Schoevers RA Oude Voshaar RC. Prevalence of frailty in community-dwelling older persons: a systematic review. J Am Geriatr Soc. (2012) 60:1487–92. 10.1111/j.1532-5415.2012.04054.x 22881367

[3] Shamliyan T Talley KM Ramakrishnan R Kane RL. Association of frailty with survival: a systematic literature review. Ageing Res Rev. (2013) 12:719–36. 10.1016/j.arr.2012.03.001 22426304

[4] Takechi H Sugihara Y Kokuryu A Nishida M Yamada H Arai H. Both conventional indices of cognitive function and frailty predict levels of care required in a long-term care insurance program for memory clinic patients in Japan. Geriatr Gerontol Int. (2012) 12:630–6. 10.1111/j.1447-0594.2011.00828.x 22300175

[5] Kulmala J Nykanen I Manty M Hartikainen S. Association between frailty and dementia: a population-based study. Gerontology. (2014) 60:16–21. 10.1159/000353859 23970189

[6] Grande G Haaksma ML Rizzuto D Melis RJF Marengoni A Onder G. Co-occurrence of cognitive impairment and physical frailty, and incidence of dementia: systematic review and meta-analysis. Neurosci Biobehav Rev. (2019) 107:96–103. 10.1016/j.neubiorev.2019.09.001 31491474

[7] Zheng L Li G Gao D Wang S Meng X Wang C. Cognitive frailty as a predictor of dementia among older adults: a systematic review and meta-analysis. Arch Gerontol Geriatr. (2020) 87:103997. 10.1016/j.archger.2019.103997 31846833

[8] Chen LK Arai H. Physio-cognitive decline as the accelerated aging phenotype. Arch Gerontol Geriatr. (2020) 88:104051. 10.1016/j.archger.2020.104051 32278485

[9] Wu YH Liu LK Chen WT Lee WJ Peng LN Wang PN. Cognitive function in individuals with physical frailty but without dementia or cognitive complaints: results from the I-lan longitudinal aging study. J Am Med Dir Assoc. (2015) 16:899 e899-16. 10.1016/j.jamda.2015.07.013 26321467

[10] Liu LK Chen CH Lee WJ Wu YH Hwang AC Lin MH. Cognitive frailty and its association with all-cause mortality among community-dwelling older adults in taiwan: results from I-lan longitudinal aging study. Rejuvenation Res. (2018) 21:510–17. 10.1089/rej.2017.2038 29644921

[11] Chen WT Chou KH Liu LK Lee PL Lee WJ Chen LK. Reduced cerebellar gray matter is a neural signature of physical frailty. Hum Brain Mapp. (2015) 36:3666–76. 10.1002/hbm.22870 26096356 PMC6869536

[12] Wallace LMK Theou O Darvesh S Bennett DA Buchman AS Andrew MK. Neuropathological burden and the degree of frailty in relation to global cognition and dementia. Neurology. (2020) 95:e3269–79. 10.1212/WNL.0000000000010944 32989103 PMC7836651

[13] DeTure MA Dickson DW. The neuropathological diagnosis of Alzheimer's disease. Mol Neurodegener. (2019) 14:32. 10.1186/s13024-019-0333-5 31375134 PMC6679484

[14] Theou O Stathokostas L Roland KP Jakobi JM Patterson C Vandervoort AA. The effectiveness of exercise interventions for the management of frailty: a systematic review. J Aging Res. (2011) 2011:569194. 10.4061/2011/569194 21584244 PMC3092602

[15] Ng TP Feng L Nyunt MS Feng L Niti M Tan BY. Nutritional, physical, cognitive, and combination interventions and frailty reversal among older adults: a randomized controlled trial. Am J Med. (2015) 128:1225–36 e1221. 10.1016/j.amjmed.2015.06.017 26159634

[16] Herr M Cesari M Landre B Ankri J Vellas B Andrieu S. Factors associated with changes of the frailty status after age 70: findings in the MAPT study. Ann Epidemiol. (2019) 34:65–70 e61. 10.1016/j.annepidem.2019.03.008 31005551

[17] Fried LP Ferrucci L Darer J Williamson JD Anderson G. Untangling the concepts of disability, frailty, and comorbidity: implications for improved targeting and care. J Gerontol A Biol Sci Med Sci. (2004) 59:255–263. 10.1093/gerona/59.3.M255 15031310

[18] Orme JG Reis J Herz EJ. Factorial and discriminant validity of the Center for Epidemiological Studies Depression (CES-D) scale. J Clin Psychol. (1986) 42:28–33. 3950011 10.1002/1097-4679(198601)42:1<28::aid-jclp2270420104>3.0.co;2-t

[19] Liou YM Jwo CJ Yao KG Chiang LC Huang LH. Selection of appropriate Chinese terms to represent intensity and types of physical activity terms for use in the Taiwan version of IPAQ. J Nurs Res. (2008) 16:252–63. 10.1097/01.JNR.0000387313.20386.0a 19061172

[20] Visvanathan R Yu S Field J Chapman I Adams R Wittert G. Appendicular skeletal muscle mass: development and validation of anthropometric prediction equations. J Frailty Aging. (2012) 1:147–51. 10.14283/jfa.2012.23 27093315

[21] Chang CC Kramer JH Lin KN Chang WN Wang YL Huang CW. Validating the Chinese version of the verbal learning test for screening Alzheimer's disease. J Int Neuropsychol Soc. (2010) 16:244–51. 10.1017/S1355617709991184 20003579 PMC3767760

[22] Mack WJ Freed DM Williams BW Henderson VW. Boston Naming Test: shortened versions for use in Alzheimer's disease. J Gerontol. (1992) 47:P154–8. 10.1093/geronj/47.3.P154 1573197

[23] Harrison JE Buxton P Husain M Wise R. Short test of semantic and phonological fluency: normal performance, validity and test-retest reliability. Br J Clin Psychol. (2000) 39:181–91. 10.1348/014466500163202 10895361

[24] Taylor LB. Localisation of cerebral lesions by psychological testing. Clin Neurosurg. (1969) 16:269–87. 10.1093/neurosurgery/16.CN_suppl_1.269 5811709

[25] Rouleau I Salmon DP Butters N Kennedy C McGuire K. Quantitative and qualitative analyses of clock drawings in Alzheimer's and Huntington's disease. Brain Cogn. (1992) 18:70–87. 10.1016/0278-2626(92)90112-Y 1543577

[26] Mok EH Lam LC Chiu HF. Category verbal fluency test performance in chinese elderly with Alzheimer's disease. Dement Geriatr Cogn Disord. (2004) 18:120–4. 10.1159/000079190 15211065

[27] Chen TB Lin CY Lin KN Yeh YC Chen WT Wang KS. Culture qualitatively but not quantitatively influences performance in the Boston naming test in a chinese-speaking population. Dement Geriatr Cogn Dis Extra. (2014) 4:86–94. 10.1159/000360695 24847347 PMC4024970

[28] Wang P Shi L Zhao Q Hong Z Guo Q. Longitudinal changes in clock drawing test (CDT) performance before and after cognitive decline. PLoS ONE. (2014) 9:e97873. 10.1371/journal.pone.0097873 24874454 PMC4038629

[29] Zhang X Lv L Min G Wang Q Zhao Y Li Y. Overview of the complex figure test and its clinical application in neuropsychiatric disorders, including copying and recall. Front Neurol. (2021) 12:680474. 10.3389/fneur.2021.680474 34531812 PMC8438146

[30] Chen LK Lee WJ Peng LN Liu LK Arai H Akishita M. Recent advances in sarcopenia research in Asia: 2016 update from the Asian Working Group for sarcopenia. J Am Med Dir Assoc. (2016) 17:767 e761–7. 10.1016/j.jamda.2016.05.016 27372539

[31] Sun Y Lee HJ Yang SC Chen TF Lin KN Lin CC. A nationwide survey of mild cognitive impairment and dementia, including very mild dementia, in Taiwan. PLoS ONE. (2014) 9:e100303. 10.1371/journal.pone.0100303 24940604 PMC4062510

[32] Yang CP Yang WS Wong YH Wang KH Teng YC Chang MH. Muscle atrophy-related myotube-derived exosomal microRNA in neuronal dysfunction: targeting both coding and long noncoding RNAs. Aging Cell. (2020) 19:e13107. 10.1111/acel.13107 32233025 PMC7253071

[33] Nishita Y Nakamura A Kato T Otsuka R Iwata K Tange C. Links between physical frailty and regional gray matter volumes in older adults: a voxel-based morphometry study. J Am Med Dir Assoc. (2019) 20:1587–92 e1587. 10.1016/j.jamda.2019.09.001 31685397

[34] Malek Rivan NF Shahar S Rajab NF Singh DKA Din NC Hazlina M. Cognitive frailty among Malaysian older adults: baseline findings from the LRGS TUA cohort study. Clin Interv Aging. (2019) 14:1343–52. 10.2147/CIA.S211027 31413555 PMC6663036

[35] Rivan NFM Shahar S Rajab NF Singh DKA Che Din N Mahadzir H. Incidence and predictors of cognitive frailty among older adults: a community-based longitudinal study. Int J Environ Res Public Health. (2020) 17:1547. 10.3390/ijerph17051547 32121194 PMC7084438

[36] JacobsJMCohenAEin-MorEMaaraviYStessmanJ. Frailty, cognitive impairment and mortality among the oldest old. J Nutr Health Aging. (2011) 15:678–82. 10.1007/s12603-011-0096-321968864

[37] Fougere B Daumas M Lilamand M Sourdet S Delrieu J Vellas B. Association between frailty and cognitive impairment: cross-sectional data from toulouse frailty day hospital. J Am Med Dir Assoc. (2017) 18:990 e991–5. 10.1016/j.jamda.2017.06.024 28797589

[38] Shimada H Doi T Lee S Makizako H Chen LK Arai H. Cognitive frailty predicts incident dementia among community-dwelling older people. J Clin Med. (2018) 7:250. 10.3390/jcm7090250 30200236 PMC6162851

[39] Gray SL Anderson ML Hubbard RA LaCroix A Crane PK McCormick W. Frailty and incident dementia. J Gerontol A Biol Sci Med Sci. (2013) 68:1083–90. 10.1093/gerona/glt013 23419778 PMC3738027

[40] Chu NM Bandeen-Roche K Tian J Kasper JD Gross AL Carlson MC. Hierarchical development of frailty and cognitive impairment: clues into etiological pathways. J Gerontol A Biol Sci Med Sci. (2019) 74:1761–70. 10.1093/gerona/glz134 31120105 PMC6777087

[41] KelaiditiECesariMCanevelliMvan KanGAOussetPJGillette-GuyonnetS. Cognitive frailty: rational and definition from an (I.A.N.A./I.A.G.G.) international consensus group. J Nutr Health Aging. (2013) 17:726–34. 10.1007/s12603-013-0367-224154642

[42] Panza F Solfrizzi V Barulli MR Santamato A Seripa D Pilotto A. Cognitive frailty: a systematic review of epidemiological and neurobiological evidence of an age-related clinical condition. Rejuvenation Res. (2015) 18:389–412. 10.1089/rej.2014.1637 25808052

[43] Verghese J Annweiler C Ayers E Barzilai N Beauchet O Bennett DA. Motoric cognitive risk syndrome: multicountry prevalence and dementia risk. Neurology. (2014) 83:718–26. 10.1212/WNL.0000000000000717 25031288 PMC4150127

[44] Chhetri JK Han C Dan X Ma L Chan P. Motoric cognitive risk syndrome in a chinese older adult population: prevalence and associated factors. J Am Med Dir Assoc. (2020) 21:136–7. 10.1016/j.jamda.2019.08.007 31623987

[45] Liu LK Guo CY Lee WJ Chen LY Hwang AC Lin MH. Subtypes of physical frailty: latent class analysis and associations with clinical characteristics and outcomes. Sci Rep. (2017) 7:46417. 10.1038/srep46417 28397814 PMC5387710

[46] Huang ST Tange C Otsuka R Nishita Y Peng LN Hsiao FY. Subtypes of physical frailty and their long-term outcomes: a longitudinal cohort study. J Cachexia Sarcopenia Muscle. (2020) 11:1223–31. 10.1002/jcsm.12577 32558267 PMC7567152

[47] Bohn L Zheng Y McFall GP Dixon RA. Portals to frailty? Data-driven analyses detect early frailty profiles. Alzheimers Res Ther. (2021) 13:1. 10.1186/s13195-020-00736-w 33397495 PMC7780374

[48] Michel JP Cruz-Jentoft AJ Cederholm T. Frailty, exercise and nutrition. Clin Geriatr Med. (2015) 31:375–87. 10.1016/j.cger.2015.04.006 26195097

[49] Pollack LR Litwack-Harrison S Cawthon PM Ensrud K Lane NE Barrett-Connor E. Patterns and predictors of frailty transitions in older men: the osteoporotic fractures in men study. J Am Geriatr Soc. (2017) 65:2473–9. 10.1111/jgs.15003 28873220 PMC5681371

[50] Abdelhafiz AH Sinclair AJ. Cognitive frailty in older people with type 2 diabetes mellitus: the central role of hypoglycaemia and the need for prevention. Curr Diab Rep. (2019) 19:15. 10.1007/s11892-019-1135-4 30806826

[51] Luchsinger JA Reitz C Patel B Tang MX Manly JJ Mayeux R. Relation of diabetes to mild cognitive impairment. Arch Neurol. (2007) 64:570–75. 10.1001/archneur.64.4.570 17420320

[52] Murukesu RR Singh DKA Shahar S Subramaniam P. A multi-domain intervention protocol for the potential reversal of cognitive frailty: “WE-RISE” randomized controlled trial. Front Public Health. (2020) 8:471. 10.3389/fpubh.2020.00471 33014971 PMC7495818

[53] Ponvel P Shahar S Singh DKA Ludin AFM Rajikan R Rajab NF. Multidomain intervention for reversal of cognitive frailty, towards a personalized approach (AGELESS trial): study design. J Alzheimers Dis. (2021) 82:673–87. 10.3233/JAD-201607 34092633 PMC8385532

[54] Lee JS Auyeung TW Leung J Kwok T Woo J. Transitions in frailty states among community-living older adults and their associated factors. J Am Med Dir Assoc. (2014) 15:281–6. 10.1016/j.jamda.2013.12.002 24534517

[55] Takatori K Matsumoto D. Social factors associated with reversing frailty progression in community-dwelling late-stage elderly people: an observational study. PLoS ONE. (2021) 16:e0247296. 10.1371/journal.pone.0247296 33657160 PMC7928521

[56] Travers J Romero-Ortuno R Bailey J Cooney MT. Delaying and reversing frailty: a systematic review of primary care interventions. Br J Gen Pract. (2019) 69:e61–9. 10.3399/bjgp18X700241 30510094 PMC6301364

[57] Trevisan C Veronese N Maggi S Baggio G Toffanello ED Zambon S. Factors influencing transitions between frailty states in elderly adults: the Progetto Veneto Anziani longitudinal study. J Am Geriatr Soc. (2017) 65:179–84. 10.1111/jgs.14515 27861714

[58] Sachdev PS Lipnicki DM Crawford J Reppermund S Kochan NA Trollor JN. Factors predicting reversion from mild cognitive impairment to normal cognitive functioning: a population-based study. PLoS ONE. (2013) 8:e59649. 10.1371/journal.pone.0059649 23544083 PMC3609866

[59] Malek-Ahmadi M. Reversion from mild cognitive impairment to normal cognition: a meta-analysis. Alzheimer Dis Assoc Disord. (2016) 30:324–30. 10.1097/WAD.0000000000000145 26908276

[60] Shimada H Doi T Lee S Makizako H. Reversible predictors of reversion from mild cognitive impairment to normal cognition: a 4-year longitudinal study. Alzheimers Res Ther. (2019) 11:24. 10.1186/s13195-019-0480-5 30867057 PMC6416893

[61] Chen LK Hwang AC Lee WJ Peng LN Lin MH Neil DL. Efficacy of multidomain interventions to improve physical frailty, depression and cognition: data from cluster-randomized controlled trials. J Cachexia Sarcopenia Muscle. (2020) 11:650–662. 10.1002/jcsm.12534 32134208 PMC7296266

[62] Coelho-Junior HJ Calvani R Picca A Goncalves IO Landi F Bernabei R. Protein-related dietary parameters and frailty status in older community-dwellers across different frailty instruments. Nutrients. (2020) 12:508. 10.3390/nu12020508 32079345 PMC7071300

[63] Liang CK Lee WJ Hwang AC Lin CS Chou MY Peng LN. Efficacy of multidomain intervention against physio-cognitive decline syndrome: a cluster-randomized trial. Arch Gerontol Geriatr. (2021) 95:104392. 10.1016/j.archger.2021.104392 33765656

[64] Kivipelto M Solomon A Ahtiluoto S Ngandu T Lehtisalo J Antikainen R. The finnish geriatric intervention study to prevent cognitive impairment and disability (FINGER): study design and progress. Alzheimers Dement. (2013) 9:657–65. 10.1016/j.jalz.2012.09.012 23332672

[65] Mantovani E Zucchella C Schena F Romanelli MG Venturelli M Tamburin S. Towards a redefinition of cognitive frailty. J Alzheimers Dis. (2020) 76:831–43. 10.3233/JAD-200137 32568197 PMC7504985

