# Supplementary material for: The Flexibility of Physio-Cognitive Decline Syndrome: A Longitudinal Cohort Study
Source: Front Public Health. 2022 Jun 6;10:820383. doi: 10.3389/fpubh.2022.820383 (PMC9207309; doi:10.3389/fpubh.2022.820383)
Supplement: Supplementary file 1 [file Data_Sheet_1.docx]

Supplementary Material

## Supplementary Figures

**Supplemental figure 1. The cumulative probability (95% confidence interval) of transitions between four groups at 2.5 years of follow-up.**

**
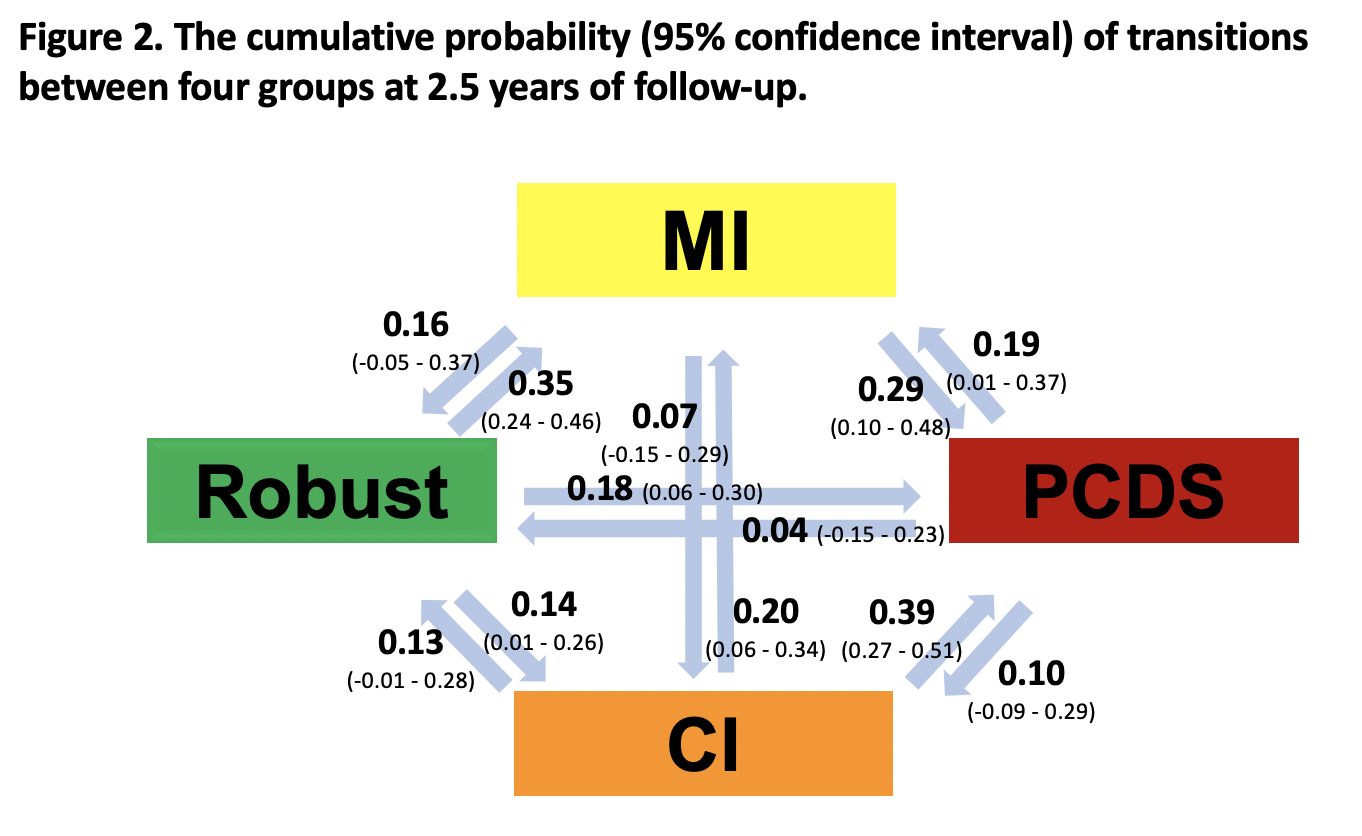
**

CI = cognitive impairment; MI = mobility impairment; PCDS = physio-cognitive decline syndrome.

*95% confidence intervals by transition: Robust to MI (95% CI = 0.24–0.46); Robust to CI (95% CI =0.01–0.26); Robust to PCDS (95% CI = 0.06–0.30); MI to Robust (95% CI = -0.05–0.37); MI to CI (95% CI = -0.15–0.29); MI to PCDS (95% CI = 0.10–0.48); CI to Robust (95% CI = -0.01–0.28); CI to MI (95% CI = 0.06–0.34); CI to PCDS (95% CI = 0.27–0.51); PCDS to Robust (95% CI = -0.15–0.23); PCDS to MI (95% CI = 0.01–0.37); PCDS to CI (95% CI = -0.09–0.29)

## Supplementary Tables

**Supplemental table 1. Baseline characteristics of participants who received or loss follow-up group**

|  | Follow-up | Loss follow-up | Total | p-value |
| --- | --- | --- | --- | --- |
| N | 531 | 692 | 1223 |  |
| Sex(F) | 46% | 53% | 50% | 0.423 |
| Age | 64.46 ± 8.58 | 61.61 ± 8.82 | 62.85 ± 8.60 | 0.001 |
| Education | 5.88 ± 4.72 | 7.67 ± 5.08 | 6.89 ± 5.01 | 0.001 |
| Weight | 62.79 ± 11.03 | 62.17 ± 10.48 | 62.44 ± 10.59 | 0.621 |
| Height | 159.03 ± 8.00 | 159.26 ± 7.97 | 159.16 ± 7.90 | 0.319 |
| Walking speed | 1.49 ± 0.44 | 1.68 ± 0.46 | 1.60 ± 0.46 | 0.001 |
| Grip strength | 29.44 ± 9.41 | 28.93 ± 9.55 | 29.15 ± 9.43 | 0.346 |
| CVVLT | 6.79 ± 2.14 | 6.56 ± 2.09 | 6.66 ± 2.03 | 0.062 |
| BNT | 9.51 ± 2.93 | 14.69 ± 2.79 | 12.44 ± 2.46 | 0.001 |
| VFT | 14.81 ± 4.66 | 14.93 ± 4.76 | 14.88 ± 4.77 | 0.653 |
| CFT | 30.86 ± 5.95 | 30.68 ± 7.00 | 30.76 ± 6.74 | 0.632 |
| CDT | 7.28 ± 2.59 | 8.08 ± 2.53 | 7.73 ± 2.48 | 0.001 |
| MMSE | 26.15 ± 3.44 | 26.50 ± 3.50 | 26.35 ± 3.37 | 0.080 |

BNT, Boston Naming Test; CDT, Clock Drawing Test; CFT, Taylor Complex Figure Test; CVVLT, Chinese Version Verbal Learning Test; MMSE, Mini-Mental State Examination; VFT, Verbal Fluency Test

p value showed significance (p < .05) between follow-up group and loss follow-up group

**Supplemental table 2. Comparisons of baseline characteristics among four groups transited from baseline robust participants (n = 206)**

|  | Baseline: Robust | | | | p-value |
| --- | --- | --- | --- | --- | --- |
| 2.5 years of follow-up | Robust | MI | CI | PCDS |  |
| n | 69 | 73 | 28 | 36 |  |
| Sex(F) | 43% | 51% | 32% | 42% | 0.401 |
| Age | 62.16 ± 7.41 | 61.03 ± 7.72 | 66.10 ± 7.91^b^ | 63.39 ± 9.34 | 0.029 |
| Education | 8.15 ± 5.58 | 6.60 ± 4.48 | 6.11 ± 4.96^b^ | 5.39 ± 3.58^c^ | 0.033 |
| Weight | 65.39 ± 10.52 | 62.72 ± 11.55 | 62.36 ± 10.63 | 64.51 ± 12.76 | 0.462 |
| Height | 162.48 ± 8.40 | 159.27 ± 7.95 | 161.14 ± 7.72 | 158.68 ± 8.68 | 0.058 |
| Walking- speed | 1.75 ± 0.41 | 1.59 ± 0.39^a^ | 1.54 ± 0.27^b^ | 1.64 ± 0.51 | 0.062 |
| Grip- strength | 34.40 ± 8.63 | 31.46 ± 9.49 | 33.43 ± 7.34 | 32.54 ± 9.45 | 0.269 |
| CVVLT | 7.93 ± 1.02 | 8.01 ± 1.09 | 7.14 ± 1.30^b^ | 7.08 ± 1.20^c^ | 0.001 |
| BNT | 11.26 ± 2.31 | 11.08 ± 2.40 | 10.39 ± 2.48 | 10.33 ± 2.29 | 0.151 |
| VFT | 17.93 ± 4.03 | 16.30 ± 4.82 | 16.57 ± 4.95^b^ | 14.97 ± 3.71^c^ | 0.010 |
| CFT | 33.62 ± 2.95 | 33.01 ± 3.46 | 32.36 ± 4.46 | 32.38 ± 3.24 | 0.222 |
| CDT | 8.93 ± 1.40^c^ | 8.41 ± 1.71 | 8.39 ± 1.57 | 8.17 ± 1.54 | 0.071 |
| MMSE | 28.03 ± 2.31 | 27.32 ± 2.85 | 26.75 ± 2.29^b^ | 26.25 ± 2.73^c^ | 0.010 |
| ASM | 19.60 ± 4.37 | 18.11 ± 4.40^a^ | 19.11 ± 3.88 | 18.68 ± 4.28 | 0.231 |
| HTN | 36% | 38% | 29% | 53% | 0.228 |
| DM | 13% | 19% | 21% | 19% | 0.691 |
| HLD | 7% | 16% | 11% | 11% | 0.401 |
| CAD | 1% | 4% | 4% | 0% | 0.542 |

ASM, appendicular skeletal muscle mass index; BNT, Boston Naming Test; CAD, cardiovascular disease; CDT, Clock Drawing Test; CFT, Taylor Complex Figure Test; CI, cognitive impairment; CVVLT, Chinese Version Verbal Learning Test; DM, diabetes mellitus; HLD, hyperlipidemia; HTN, hypertension; MI, mobility impairment; MMSE, Mini-Mental State Examination; PCDS, physio-cognitive decline syndrome; VFT, Verbal Fluency Test

*Significant after posthoc analyses between groups. a: significance between MI and robust group; b: significance between CI and robust group; c: significance between PCDS and robust group; d: significance between MI and CI; e: significance between MI and PCDS; f: significance between CI and PCDS.

Chi-square analysis using Tukey’ test with corrected p value, significantly (p < .05)

**Supplemental table 3. Comparisons of baseline characteristics among four groups transited from baseline MI participants (n = 75)**

|  | Baseline: MI | | | | p-value |
| --- | --- | --- | --- | --- | --- |
| 2.5 years of follow-up | Robust | MI | CI | PCDS |  |
| n | 12 | 36 | 5 | 22 |  |
| Sex(F) | 33% | 39% | 60% | 64% | 0.211 |
| Age | 62.19 ± 6.63 | 60.01 ± 6.79 | 64.14 ± 6.10 | 68.69 ± 9.95^e^ | 0.001 |
| Education | 7.58 ± 4.48 | 6.89 ± 5.05 | 6.40 ± 3.29 | 3.23 ± 4.00^e^ | 0.019 |
| Weight | 66.68 ± 8.56 | 62.16 ± 10.05 | 64.70 ± 8.75 | 57.07 ± 10.96^e^ | 0.051 |
| Height | 158.86 ± 8.19 | 158.32 ± 7.37 | 161.78 ± 8.37 | 154.66 ± 7.50 | 0.154 |
| Walking- speed | 1.33 ± 0.47 | 1.33 ± 0.39 | 1.32 ± 0.45 | 1.06 ± 0.28^e^ | 0.050 |
| Grip- strength | 32.17 ± 7.65 | 27.28 ± 9.38 | 27.40 ± 9.24 | 20.48 ± 7.71^e^ | 0.002 |
| CVVLT | 8.33 ± 0.98 | 7.72 ± 1.19 | 7.20 ± 1.10 | 7.23 ± 1.19 | 0.052 |
| BNT | 10.42 ± 1.68 | 11.06 ± 1.93 | 9.00 ± 2.24^d^ | 9.00 ± 1.75^e^ | 0.001 |
| VFT | 16.00 ± 4.57 | 16.92 ± 3.83 | 14.20 ± 1.30 | 13.82 ± 4.03^e^ | 0.030 |
| CFT | 34.21 ± 2.10 | 32.25 ± 4.47 | 34.00 ± 1.58 | 29.52 ± 4.72 | 0.010 |
| CDT | 8.83 ± 1.03 | 8.50 ± 1.48 | 6.80 ± 0.84^d^ | 7.23 ± 2.18^e^ | 0.012 |
| MMSE | 26.92 ± 2.61 | 28.06 ± 1.79 | 27.20 ± 1.79 | 25.68 ± 2.68^e^ | 0.003 |
| ASM | 18.94 ± 3.47 | 18.23 ± 3.79 | 18.99 ± 4.57 | 14.76 ± 3.26^e^ | 0.002 |
| HTN | 42% | 50% | 40% | 36% | 0.788 |
| DM | 17% | 25% | 0% | 23% | 0.616 |
| HLD | 0% | 17% | 0% | 14% | 0.393 |
| CAD | 0% | 0% | 0% | 5% | 0.504 |

ASM, appendicular skeletal muscle mass index; BNT, Boston Naming Test; CAD, cardiovascular disease; CDT, Clock Drawing Test; CFT, Taylor Complex Figure Test; CI, cognitive impairment; CVVLT, Chinese Version Verbal Learning Test; DM, diabetes mellitus; HLD, hyperlipidemia; HTN, hypertension; MI, mobility impairment; MMSE, Mini-Mental State Examination; PCDS, physio-cognitive decline syndrome; VFT, Verbal Fluency Test

*Significant after posthoc analyses between groups. a: significance between MI and robust group; b: significance between CI and robust group; c: significance between PCDS and robust group; d: significance between MI and CI; e: significance between MI and PCDS; f: significance between CI and PCDS.

Chi-square analysis using Tukey’ test with corrected p value, significantly (p < .05)

**Supplemental table 4. Comparisons of baseline characteristics among four groups transited from baseline CI participants (n = 154)**

|  | Baseline: CI | | | | p-value |
| --- | --- | --- | --- | --- | --- |
| 2.5 years of follow-up | Robust | MI | CI | PCDS |  |
| n | 21 | 32 | 42 | 59 |  |
| Sex(F) | 48% | 56%^d^ | 31% | 46% | 0.171 |
| Age | 61.74 ± 6.77^b^ | 60.31 ± 6.61^d^ | 69.41 ± 7.87 | 68.00 ± 8.53 | 0.001 |
| Education | 6.24 ± 3.35 | 6.44 ± 4.30 | 6.36 ± 4.97 | 4.61 ± 4.23 | 0.124 |
| Weight | 63.58 ± 12.27 | 62.89 ± 11.52 | 63.34 ± 9.36 | 63.81 ± 8.95 | 0.981 |
| Height | 161.57 ± 8.87 | 158.78 ± 7.91 | 160.79 ± 5.49 | 158.80 ± 6.92 | 0.266 |
| Walking- speed | 1.58 ± 0.32 | 1.60 ± 0.53 | 1.62 ± 0.45 | 1.49 ± 0.38 | 0.452 |
| Grip- strength | 30.93 ± 9.31 | 32.23 ± 9.63 | 30.87 ± 5.70 | 28.65 ± 7.37 | 0.176 |
| CVVLT | 6.33 ± 2.29 | 7.19 ± 1.57^d^ | 5.55 ± 2.29 | 5.66 ± 2.68 | 0.011 |
| BNT | 10.38 ± 2.50^b^ | 9.09 ± 2.52 | 8.02 ± 3.48 | 7.85 ± 2.60 | 0.003 |
| VFT | 13.81 ± 4.67 | 14.56 ± 5.40 | 13.19 ± 3.49 | 12.36 ± 4.14 | 0.134 |
| CFT | 32.10 ± 4.21 | 31.80 ± 5.32 | 29.33 ± 6.76 | 27.95 ± 6.48 | 0.010 |
| CDT | 7.29 ± 2.49 | 7.03 ± 2.51 | 6.83 ± 2.56 | 5.66 ± 2.86^f^ | 0.028 |
| MMSE | 26.38 ± 3.26 | 26.81 ± 3.06 | 25.67 ± 3.53 | 24.92 ± 3.44 | 0.062 |
| ASM | 19.10 ± 4.92 | 17.99 ± 3.93 | 18.88 ± 3.10 | 18.22 ± 3.63 | 0.624 |
| HTN | 48% | 22% | 34% | 57%^f^ | 0.013 |
| DM | 10% | 16% | 7% | 29%^f^ | 0.031 |
| HLD | 5% | 9% | 2% | 16% | 0.144 |
| CAD | 10% | 6% | 5% | 10% | 0.909 |

ASM, appendicular skeletal muscle mass index; BNT, Boston Naming Test; CAD, cardiovascular disease; CDT, Clock Drawing Test; CFT, Taylor Complex Figure Test; CI, cognitive impairment; CVVLT, Chinese Version Verbal Learning Test; DM, diabetes mellitus; HLD, hyperlipidemia; HTN, hypertension; MI, mobility impairment; MMSE, Mini-Mental State Examination; PCDS, physio-cognitive decline syndrome; VFT, Verbal Fluency Test

*Significant after posthoc analyses between groups. a: significance between MI and robust group; b: significance between CI and robust group; c: significance between PCDS and robust group; d: significance between MI and CI; e: significance between MI and PCDS; f: significance between CI and PCDS.

Chi-square analysis using Tukey’ test with corrected p value, significantly (p < .05)
